# Epidemiology and clinical spectrum of leishmaniasis due to *Leishmania infantum* in Tunisia: Current status, challenges, and perspectives

**DOI:** 10.1371/journal.pntd.0014063

**Published:** 2026-03-12

**Authors:** Najla Chargui, Ahmad Amro, Hamouda Babba, Najoua Haouas

**Affiliations:** 1 Laboratory of Medical and Molecular Parasitology‑Mycology, Department of Clinical Biology B, Faculty of Pharmacy, University of Monastir, Monastir, Tunisia; 2 Faculty of Pharmacy, Al-Quds University, East Jerusalem, Palestine; FIOCRUZ Bahia: Instituto Goncalo Moniz, BRAZIL

## Abstract

**Background:**

This review focuses on leishmaniasis caused by *Leishmania (L.) infantum* in Tunisia, a vector-borne parasitic disease transmitted through the bite of infected female sandflies. Leishmaniasis manifests as a spectrum of clinical forms, ranging from benign cutaneous lesions, to a severe and potentially fatal visceral form. In Tunisia, *L. infantum* is the etiological agent of visceral leishmaniasis (VL) and cutaneous leishmaniasis (CL). While CL typically manifests as a single, small facial lesion, atypical forms are sometimes observed. VL primarily affects children under the age of five and immunocompromised individuals, although an increasing number of cases have been reported in immunocompetent adults in recent years. Although neglected, leishmaniasis is an emerging and growing public health concern in Tunisia, particularly due to the increasing incidence of VL among adults and potential spread of both CL and VL to previously non-endemic areas. This expansion is demonstrated by the fact that *L. infantum* has a geographical distribution mainly in the humid, sub-humid and semi-arid regions of the north, but gradually spreading towards the central and southern parts of the country.

**Methodology/principal findings:**

This literature review was conducted through a systematic search of PubMed, Scopus, and Google Scholar for studies published between 1904 and 2024, focusing on the clinical, epidemiological, molecular, and ecological aspects of *L. infantum* in Tunisia. In addition to its clinical variability, *L. infantum* presents a biochemical variability with three isoenzymatic variants (zymodemes) identified in Tunisia: MON-1 (predominantly associated with VL), MON-24 (predominantly associated with CL), and MON-80 (implicated in both forms). Our review found that VL remains highly endemic in northern Tunisia but has expanded southward in recent decades, while cutaneous cases due to *L. infantum* are increasingly recognized. Isoenzymatic and molecular studies confirm the predominance of the MON-1 zymodeme, with sporadic detection of MON-24 and MON-80. Domestic dogs remain the main reservoir, and *Phlebotomus (P.) perniciosus* is the principal vector for VL, though other *Phlebotomus* species have been implicated in CL transmission. These findings highlight the importance of integrating molecular tools alongside classical isoenzyme methods for a better understanding of parasite dynamics and epidemiological monitoring.

**Conclusions/significance:**

The transmission cycle of *L. infantum* is not fully elucidated, but domestic dogs and *P. perniciosus* are considered the primary reservoir and vector for VL, respectively, while, other potential mammalian hosts and sandflies vectors were suspected for CL. Comparative data from Algeria, Morocco, Libya, and southern Europe suggest both common patterns and local specificities in *L. infantum* transmission, underscoring the importance of regional collaboration. The epidemiological and clinical complexity of *L. infantum*, together with its expanding geographic distribution in Tunisia, underscores the need for further integrated research to clarify transmission cycles and implement effective prevention and control strategies.

## Introduction

Leishmaniasis is a vector-borne disease caused by a protozoan parasite of the genus *Leishmania*, which transmitted to humans by the bite of infected female sandflies. The progression of infection is determined by the specific parasite-host cell interaction. Distinct balances between parasite replication dynamics and host immune responses across species [1–3] account for the broad spectrum of clinical manifestations in humans, which range from localized cutaneous lesions to severe and potentially fatal visceral disease.

Three *Leishmania* species coexist in Tunisia: *Leishmania (L.) major*, *L. killicki*, and *L. infantum*. *L. major* is the predominant species, occurring mainly in central and southern Tunisia. *L. infantum* and *L. killicki* occur sporadically, mainly in the north and south, respectively. Infections with *Leishmania* species manifest in various clinical forms. While *L. major* and *L. killicki* cause exclusively self-healing cutaneous leishmaniasis (CL), *L. infantum* is responsible for distinct clinical manifestations, ranging from cutaneous leishmaniasis to visceral leishmaniasis (VL), a potentially fatal disease if untreated. Although *L. infantum* primarily causes visceral leishmaniasis and sporadic cutaneous leishmaniasis, a recent report indicates that it can also rarely cause mucosal leishmaniasis in Tunisia [4]. This finding highlights the broader clinical spectrum of *L. infantum* infections and underscores the need for continued surveillance and updated epidemiological assessments.

In addition, leishmaniasis caused by *L. infantum* has long been restricted to the northern regions of Tunisia. In recent years, both human and canine leishmaniasis were spread to other areas [5–9]. It is therefore necessary to update the status of leishmaniasis due to *L. infantum* in order to monitor the extent of the disease in Tunisia.

Moreover, leishmaniasis is among the highest-priority neglected vector-borne tropical diseases worldwide [10,11]. Epidemiological studies of these parasitoses aim to identify the key components of the parasite cycle, including the vertebrate host (reservoir), the invertebrate host (vector), and the *Leishmania* parasite.

So far, the dog is the unique proven reservoir host for *L. infantum*, and *P. perniciosus* is the vector. However, recent evidence suggests that *L. infantum* has multiple transmission pathways involving various species as reservoir and vector hosts. The diversity of *Leishmania* species, vectors, and reservoirs contributes to the complex epidemiology of the disease. In addition, environmental and climatic factors, including temperature, humidity, and land use, significantly influence sandfly distribution and incidence of leishmaniasis [12].

## Methods

This literature review was conducted to examine leishmaniasis due to *L. infantum* in Tunisia focusing on clinical and epidemiological parameters. Searches were performed in PubMed, Google Scholar, and Scopus, covering publications from 1904 to 2024, using keywords such as “leishmaniasis,” “*Leishmania infantum*,” “Tunisia,” “clinical forms,” “epidemiology,” “reservoir,” and “vector.” Inclusion criteria encompassed original research articles, reviews, and peer-reviewed studies that addressed *L. infantum* in Tunisia, as well as comparative analyses with other Mediterranean countries. Only studies published in English or French were considered. Exclusion criteria included papers unrelated to the topic (e.g., focused mainly on immunology or without available full text). We identified 153 records from PubMed, 182 from Scopus, and 255 from Google Scholar. After removal of duplicates, 398 records remained and were screened by title and abstract, leading to the exclusion of 258 records based on our exclusion criteria. About 140 full-text articles were assessed for eligibility, of which 58 were excluded, mainly because they lacked clinical or epidemiological data or had no accessible full text. Finally, around 82 studies were included in the qualitative synthesis. This approach was designed to provide a comprehensive update of knowledge about leishmaniasis due to *L. infantum* in Tunisia.

### 1. Visceral leishmaniasis in Tunisia

#### 1.1. History and impact.

In Tunisia, VL is an old disease. The first documented case was described in Tunis in 1904 [13]. Although VL is less common than CL, its public health impact is significant. Prior to 2012, the annual incidence in Tunisia was estimated at approximately 89 cases [14]. However, over the past decade, the incidence of VL has increased, and its geographical range has expanded, particularly in central and northern regions, resulting in an increase in both disease burden and healthcare costs [5,15,16]. The expansion of VL foci is linked to environmental and ecological changes that favor the coexistence of reservoir hosts (mainly dogs) and competent sandfly vectors [17]. VL represents a major public health concern in Tunisia, not only because of its clinical severity but also due to its socioeconomic impact, particularly in rural areas where healthcare resources are limited. Timely diagnosis, effective treatment, and vector control measures remain essential for reducing morbidity and mortality.

#### 1.2. Clinical forms.

In Tunisia, VL is predominantly known for its Mediterranean infantile form, developing in approximately 95% of cases in children under five years of age [18]. The common symptoms of VL are splenomegaly (97.9%), fever (79.9%), and hepatomegaly (47.3%). [19]. Nevertheless, adult cases were increasingly reported [20,21]. Since 2000, adult cases have risen to nearly 10% [22]. This shift in the epidemiological profile is primarily attributed to factors leading to immunosuppression, including viral infections such as Human Immunodeficiency Virus (HIV) and iatrogenic causes (e.g., corticosteroids, immunosuppressants, and anti-mitotic drugs) [21,23]. *L. infantum* acts as an opportunistic parasite favored by any cellular immunity deficit. In Tunisia, however, VL-HIV co-infection is rare [24,25].

#### 1.3. Geographical breakdown.

**Fig 1:**
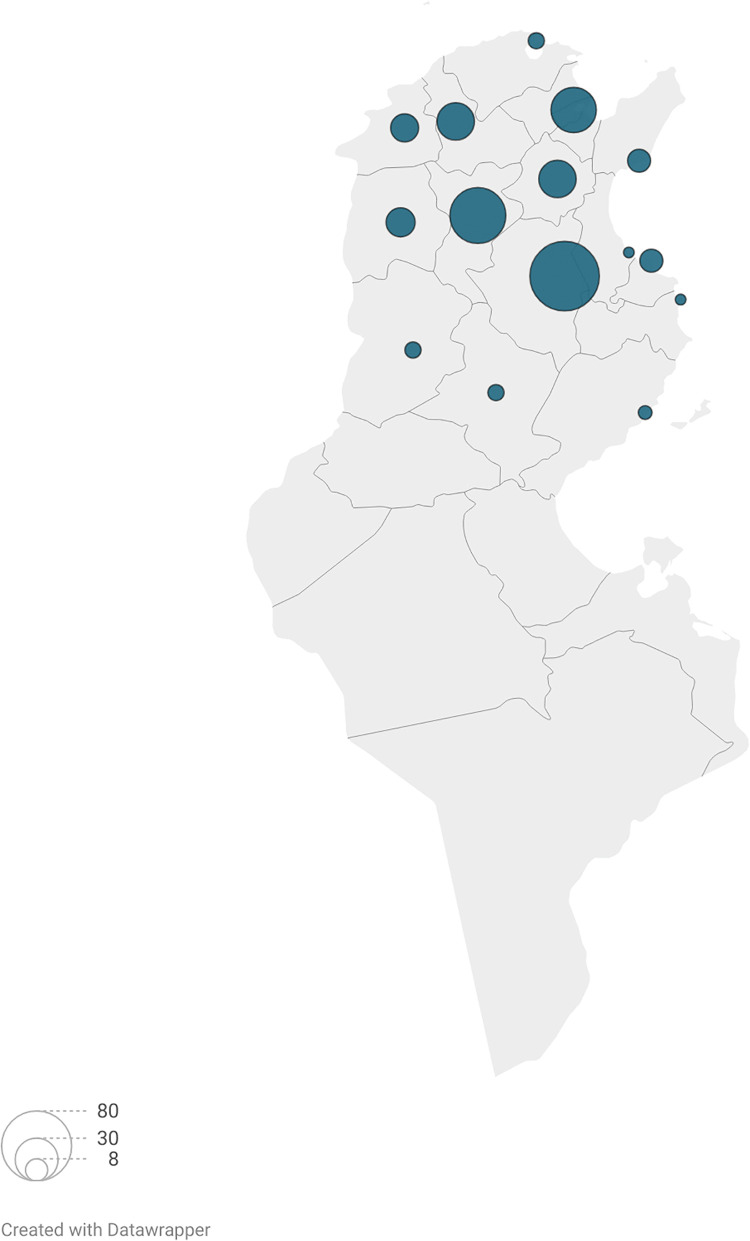
Geographical distribution of human visceral leishmaniasis due to *Leishmania infantum* cases identified by isoenzymatic method between 2001 and 2008 in Tunisia [7,21,22,25,26–28]. Map created with Datawrapper using Natural Earth public domain basemap (https://www.naturalearthdata.com). The numbers next to the circles indicate the number of cases.

In Tunisia, VL is distributed across humid, sub-humid, semi-arid and arid bioclimatic zones. It was historically endemic and widely distributed in the northern regions of the country before progressively expanding towards the center (Fig 1). Several Northern provinces are known to be endemic for this form of leishmaniasis [7,15,25,29,30]. In addition, autochthonous cases have been reported in central provinces [5,7,25,31–34].

This expansion is likely driven by a combination of environmental, climatic, and anthropogenic factors, including increased human and animal mobility, agricultural development, and changes in land use that favor sandfly vector proliferation and reservoir host movement [17,35]. Such ecological shifts pose challenges for disease control programs, particularly in newly affected areas where surveillance, diagnostic infrastructure, and clinical expertise are often limited.

### 2. Cutaneous leishmaniasis due to *L. infantum* in Tunisia

#### 2.1. History and impact.

*L. infantum* is primarily recognized as viscerotropic species responsible for VL in both the Old and New Worlds. However, cutaneous leishmaniasis (CL) caused by *L. infantum* was described for the first time in Tunisia in the early 20th century [36]. Since then, sporadic CL cases have been reported with only a few cases each year. In Tunisia, the average annual incidence is estimated at approximately 30 cases [37]. These cases often occur in areas where VL is also endemic, indicating overlapping transmission cycles.

#### 2.2. Clinical forms.

The classic cutaneous lesion caused by *L. infantum* is usually localized and is characterized by a single, small, ulcerated or lupoid facial lesion, which may persist for several months to up to three years [38]. Unusual and atypical presentations, such as diffuse cutaneous leishmaniasis and lesions mimicking other dermatological conditions, have also been documented, highlighting the need for accurate differential diagnosis [39,40]

#### 2.3. Geographical breakdown.

**Fig 2:**
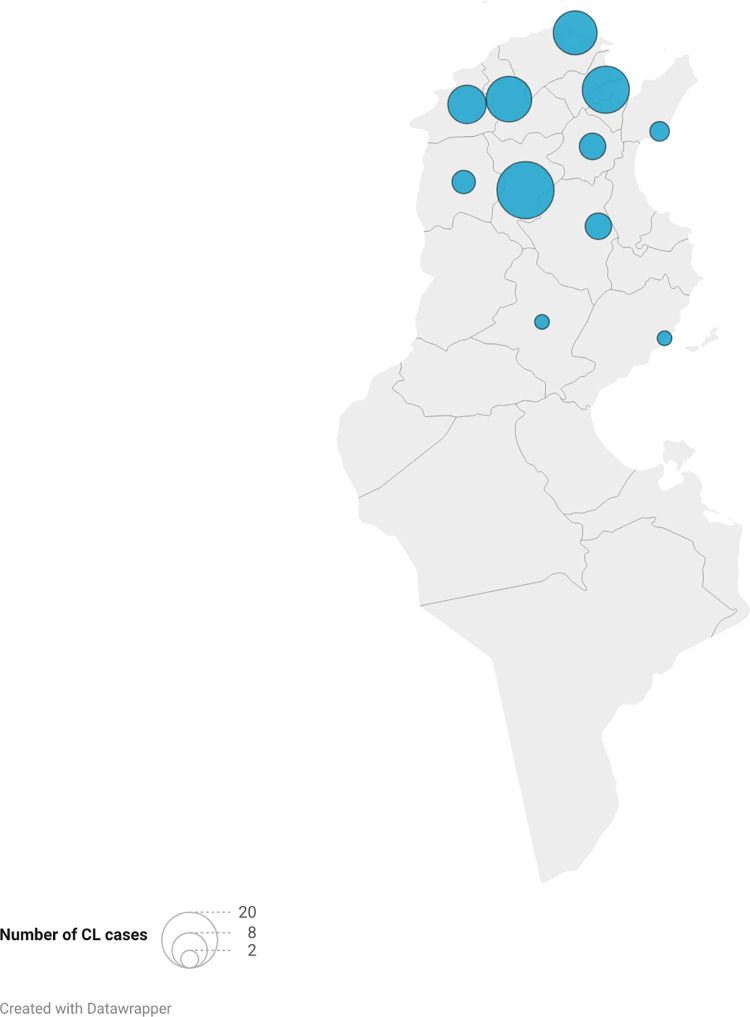
Geographical distribution of cutaneous leishmaniasis due to *Leishmania infantum* cases identified by isoenzymatic method between 2001 and 2008 in Tunisia [26,28,37,42,43]. Map created with Datawrapper using Natural Earth public domain basemap (https://www.naturalearthdata.com). The numbers next to the circles indicate the number of cases.

CL due to *L. infantum* is sporadically distributed in Tunisia, occurring in humid, sub-humid, and semi-arid bioclimatic conditions. This parasite is predominantly found in the north as the VL, supporting its dual role in visceral and cutaneous disease [37,41]. However, molecular and epidemiological surveys have indicated a progressive southward expansion; currently, *L. infantum* is also detected in central regions (Fig 2) [7,42,44]. Microsatellite analyses from Morocco and Algeria suggest possible genetic flow between neighboring foci, which may have implications for future spread in Tunisia [45]. Clinical, geographical repartition and Main challenges about *L. infantum* in Tunisia were summarized in Table 1.

**Table 1:** Clinical spectrum and geographical distribution of *Leishmania infantum* infections in Tunisia.

**Parameter**	Visceral leishmaniasis (VL)	Cutaneous leishmaniasis (CL)
**First description**	1904 [13]	1918 [36]
**Clinical aspects**	mostly infantile form [18], rarely adults [20]	mostly a single, small, ulcerated or lupoid lesion on the face [38,44] with rare atypical forms [41,46]
**Main endemic areas**	North Tunisia: humid, subhumid, and semi-arid zones [7,15,25,29,30]	North Tunisia: humid, subhumid, and semi-arid zones [37,41]
**Sporadic areas**	Central Tunisia: arid and Saharan regions [5,7,25,31–33]	Central Tunisia: arid and Saharan regions [7,42,44]
**Isoenzymatic variants**	MON-1 (75.1%), MON-24 (21.4%), and MON-80 (3.5%) [21,22,25,26–28,37,42,43]	MON-24 (84.4%), MON-1 (13.1%), and MON-80 (2.5%) [21,22,25,26–28,37,42,43]
**Main challenges**	Monitoring the increasing incidence of VL among adults and potential spread of both CL and VL to previously non-endemic areas	
	Integrating molecular tools alongside classical isoenzymatic methods for understanding of parasite dynamics	

Data compiled from published epidemiological and clinical studies between 1904 and 2024. Percentages reflect case distributions reported in cited studies. All references to specific information are cited in the corresponding places within the table.

### 3. Epidemiology of *L. infantum* in Tunisia

Epidemiological studies are conducted by analyzing all parameters of the *L. infantum* transmission cycle (parasite, vector, and reservoir)

#### 3.1. Identifying the parasite.

In Tunisia, isoenzymatic analysis of *L. infantum* strains revealed three zymodemes (enzymatic variants): MON-1, MON-24, and MON-80. These zymodemes can cause both CL and VL. *L. infantum* zymodemes MON-1 and MON-80 mainly cause VL (91.3% and 72.7% of cases, respectively), while MON-24 is predominantly associated with CL (68.2%) [21,22,25,26–28,37,42,43]. Thus, there is no strict correlation between taxa and clinical appearance. These findings highlight the importance of integrating molecular tools, such as Multi-Locus Microsatellite Typing (MLMT) or genomic sequencing, alongside classical isoenzymatic methods for accurate epidemiological monitoring. While isoenzymatic analysis (zymodeme identification) remains the gold standard for species and strain designation, it has limited resolution to detect genetic recombination, hybrid strains, or population-level gene flow. Molecular methods complement this by uncovering such hidden genetic diversity and transmission dynamics, providing a more complete understanding of the parasite’s ecology and evolution, which is crucial for effective surveillance and control strategies [47].

#### 3.2. The Reservoir.

*Leishmania* parasites are maintained mostly in animal reservoirs, which allow the survival of the parasite between two seasons of transmission. The transmission cycle of *L. infantum* is primarily zoonotic, with domestic dogs serving as the main reservoir hosts for *L. infantum*, as in all Mediterranean countries [6,26,28,48]. Canine infection with *L. infantum* is important as a cause of disease in dogs and as a reservoir for human leishmaniasis. A spatial correlation was confirmed between the occurrence of human VL and the high rate of *Leishmania* infection in Tunisian dogs. The detection of *L. infantum* in dogs confirmed an increase of the prevalence and an extension of the spatial repartition [6–8,16,49].

Besides dogs, other species have also been reported to be infected by *L. infantum*. Derghal et al. detected *L. infantum* DNA in hedgehogs (*Atelerix algirus*) and rodents (*Meriones shawi*) [50]. Further investigations need to be conducted to confirm their potential role in *Leishmania* transmission.

#### 3.3. The vector.

In Tunisia, *P. perniciosus* was proven as the main vector of *L. infantum* [51], confirmed recently by uses of molecular methods [16,52–56]. *P. perniciosus* would be able to transmit both viscerotropic and dermotropic strains as supported by experimental evidence [57]. To fully understand the role of *P. perniciosus* in transmitting dermotropic *L. infantum*, further studies are needed by isolating and identifying *Leishmania* in naturally infected sandflies. Furthermore, morphological identifications must be coupled with molecular tools for precise species discrimination. This is particularly crucial given the phenotypic polymorphism observed in Tunisian *P. perniciosus* [58–60].

Alongside *P. perniciosus*, additional sandfly species may play a secondary role in the transmission life cycle of *L. infantum*, highlighting the complexity of vector ecology in the region [16,53,55,61]. For example, *L. infantum* DNA has been detected in *P. perfiliewi* [54,61], *P. longicuspis* [53,54], and *P. langeroni* [62]. Analyzing all reported cases of *L. infantum*-infected sandflies, revealed that the protozoan is most commonly detected in *P. perniciosus* (64.1%) the confirmed vector, but it has also been identified in *P. perfiliewi* (13.4%), *P. longicuspis (8.9%),* and in *P. langeroni* (3%) (Table 2). However, the incrimination of a species of sand fly as a vector of leishmaniasis should be confirmed by other parameters as a significant anthropophilic behavior, vector capacity, the simultaneous presence of the vector and the disease, and the abundance of the vector [64]. In addition, infected sandflies belonging to *Sergentomya* genus, normally associated with the transmission of reptile *Leishmania*, have been detected in *minuta* and *dreyfussi* [63]. However, experimental infestation of *S. schwetzi* proved that this phlebotomine species is refractory to human *Leishmania* species [65].

**Table 2:** Reported cases of *Leishmania infantum*-infected sandflies observed in Tunisia.

Species of Sandfly	Numbers of cases	Percentages
*P. (Larroussius) perniciosus*	43	64.1
*P. (Larroussius) perfiliewi*	9	13.4
*P. (Larroussius) longicuspis*	6	8.9
*P. (Larroussius) langeroni*	2	3
Total *P. (Larroussius)*	60	89.5
*S. minuta*	4	5
*S. dreyfussi*	3	4.4
Total *Sergentomya*	7	10.5
Total	67	100

Data derived from entomological surveys conducted between 1993 and 2023 [53–55,61,62,63]. Numbers represent total sandflies found positive for *L. infantum* DNA; percentages correspond to species-specific infection rates.

In addition, sandfly analysis is useful for investigate potential *Leishmania* reservoirs. The analysis of blood meals in sand flies aims to identify the trophic preferences of sand flies and then learn more about other potential reservoirs not yet proven. Thus, DNA from mammalian species has been detected in *P. (Larrousius) spp* blood meals. Species such as *Homo sapiens* (human), as well as domestic animals such as *Bos taurus* (cow), *Capra hircus* (goat), *Ovis aries* (sheep), *Oryctolagus cuniculus* (rabbit), and *Equus caballus* (horse) have been identified [53,54] (Table 3). Furthermore, equids and wild rabbits are potential reservoirs since infected cases have been detected worldwide [66–69]. Human would be the reservoir of cutaneous form since CL lesions can persist for up to three years. Unexpectedly, no DNA of dogs, a proven reservoir of *L*. *infantum* in Tunisia, has been found in sandfly blood meal analysis in Tunisia. The presence of broad host availability could probably explain this result in the vicinity of the traps confirming the opportunistic behavior of species of this *sub*genus. This observation highlights the importance of conducting investigations focused on identified species in blood meal of sandflies to analyze their implication in the transmission life cycle of leishmaniasis.

**Table 3:** Reported blood meal sources of *Phlebotomus (Larroussius)* spp. sandflies infected with *Leishmania infantum* in Tunisia.

Sand flies species	Blood meal typing							
	*Bos taurus*	*Homo sapiens*	*Ovis aries*	*Capra hircus*	*Equus caballus*	*Equus asinus africanus*	*Meleagris gallopavo*	Total (%)
*P. perniciosus*	19	19	4	6	0	0	0	50 (40.3%)
*P. perfiliewi*	37	10	4	1	0	0	0	56 (45.1%)
*P. longicuspis*	5	7	2	0	1	1	1	18 (14.5%)
Total (%)	51 (41.1%)	26 (21%)	10 (8%)	7 (5.6%)	1 (0.8%)	2 (1.8%)	1 (0.8%)	124

Data from molecular blood meal analyses of sandflies collected in Tunisia between 2018 and 2020 [53,54]. Values represent the number of blood meals identified per host species, with percentages indicating relative frequency.

Also, unexpected anthropophilic feeding behavior of *Sergentomya* suggests its implication in the transmission of mammalian *Leishmania* to human [63]. Table 4 summarizes epidemiological features of *L. infantum* in Tunisia.

**Table 4:** Key epidemiological parameters of the *Leishmania infantum* transmission cycle in Tunisia.

**Confirmed vector(s)**	*P. perniciosus* [51]
**Suspected vectors**	*P. perfiliewi*, *P. longicuspis, P. langeroni* [53,54,61,62]
**Known reservoirs**	Domestic dogs [48]
**suspected reservoirs**	Species infected by *L. infantum* DNA: *Atelerix algirus* (hedgehog), *Meriones shawi* (rodent) [50]
	Vertebrate species identified through the identification of the Blood meal origin of engorged *P. perniciosus* specimens: *Bos taurus* (cow), *Capra hircus* (goat), *Ovis aries* (sheep), *Oryctolagus cuniculus* (rabbit), and *Equus caballus* (horse) [53,54]
**Main challenges**	Need for further integrated research to clarify transmission cycles, identify additional vectors and reservoirs among suspected ones.

Data summarized from multiple epidemiological, molecular, and entomological studies conducted in Tunisia between 1904 and 2023. Table highlights the main confirmed and suspected vectors and reservoirs. All references to specific information are cited in the corresponding places within the table.

### 4. Comparative perspective with neighboring countries

The epidemiological situation of *L. infantum* leishmaniasis in Tunisia shares similarities with other Maghreb countries, but also presents distinctive features. In Algeria, visceral leishmaniasis remains highly endemic in the north and has also extended southwards, with *L. infantum* MON-1 being the predominant zymodeme [70,71], similar to Tunisia. However, cutaneous cases due to *L. infantum* are less frequently reported compared to Tunisia. In Morocco, *L. infantum* is responsible for both VL and sporadic CL, and multi-locus microsatellite typing studies suggest genetic flow across borders with Algeria and Tunisia [72], which may explain the spread of atypical cutaneous forms. In Libya, *L. infantum* is mainly associated with VL in coastal and sub-humid areas, but sporadic CL cases have also been described [73,74]. These comparisons highlight that *L. infantum* in North Africa shows consistent zoonotic transmission cycles with dogs as the primary reservoir and *P. perniciosus* as the main vector, but differences in clinical presentations and geographical expansion may reflect local ecological, climatic, and socio-economic factors [75].

Integrating epidemiological data from the Maghreb region is essential to understand regional parasite dynamics, anticipate future expansion under climate change scenarios, and design cross-border surveillance and control strategies.

### 5. Challenges and future perspectives: drug resistance, climate change, and co-infections

#### 5.1. Drug resistance.

Although antimonial drugs and amphotericin B remain the cornerstone treatments for visceral leishmaniasis in Tunisia, emerging evidence from the Mediterranean basin suggests decreasing efficacy of pentavalent antimonials and variable response to amphotericin formulations [76,77]. Reports from Algeria and Morocco indicate sporadic treatment failures, raising concerns that drug resistance may also emerge in Tunisia [78]. Continuous monitoring of treatment outcomes and the introduction of molecular markers of resistance are essential to guide clinical management and policy decisions.

#### 5.2. Impact of climate change.

Climate change is expected to profoundly alter the distribution of sandfly vectors in North Africa [79]. Rising temperatures, changes in humidity, and expansion of irrigated agricultural zones may create suitable habitats for *Phlebotomus* species in areas previously free of leishmaniasis. Predictive models from North Africa suggest a north-to-south expansion of *L. infantum* foci, with Tunisia being particularly vulnerable due to its diverse bioclimatic zones [79,80]. This highlights the urgent need to integrate climate data into national surveillance and vector control programs.

#### 5.3. Co-infections, particularly HIV.

While HIV co-infection with VL is still rare in Tunisia, it has been increasingly reported in other Mediterranean countries, such as Libya [81], Spain, and Italy, where *L. infantum* acts as an opportunistic pathogen in immunocompromised patients [82,83]. With the rising prevalence of HIV in North Africa, the possibility of increased VL–HIV co-infections cannot be excluded. Such cases are associated with atypical clinical presentations, higher relapses rates, and increased mortality. Enhanced clinical awareness and cross-screening for both diseases are therefore crucial.

## Conclusion

Given the complexity of *L. infantum* from both an epidemiological and clinical perspective, this taxon requires further investigation in Tunisia. Its geographical distribution is extended from the north to the center regions of the country. Also, the transmission cycle of this parasite is not entirely known; many sandflies and mammalian species are suspected of being potential vectors and reservoirs. Further comprehensive and integrated epidemiological and entomological investigations are crucial to clearly elucidate the transmission cycles of *L. infantum*, identify potential new vectors or reservoirs, and guide targeted control strategies. Moreover, continued monitoring of climatic, environmental, and human-induced changes will be essential to predict emerging foci and adapt public health interventions accordingly.

**Key learning points**

- Leishmaniasis is a vector-borne parasitic disease transmitted through the bite of infected female sandflies.
- Leishmaniasis is an emerging and growing public health concern in Tunisia, particularly due to the increasing incidence of visceral leishmaniasis among adults and potential spread of both cutaneous and visceral leishmaniais to previously non-endemic areas.
- Leishmaniasis due to *Leishmania infantum* is zoonotic, with the domestic dog confirmed as the main reservoir, while other potential mammalian hosts remain to be identified.
- *Phlebotomus perniciosus* is the only confirmed vectors, although several other sandfly species are suspected to contribute to *Leishmania infantum* transmission.

**Key papers**

- Gramiccia M, Ben-Ismail R, Gradoni L, Ben Rachid MS, Ben Said M. A *Leishmania infantum* enzymatic variant, causative agent of cutaneous leishmaniasis in north Tunisia. Transactions of the Royal Society of Tropical Medicine and Hygiene. 1991;85:370–1.
- Aoun K, Bouratbine A, Harrat Z, Guizani I, Mokni M, Ali S, et al. Données épidémiologiques et parasitologiques concernant la leishmaniose cutanée sporadique du nord tunisien Bulletin de la Societe de pathologie exotique. 2000;93(2):101–3.
- Haouas N, Gorcii M, Chargui N, Aoun K, Bouratbine A, Akrout FM, et al. Leishmaniasis in central and southern Tunisia: current geographical distribution of zymodemes. Parasite (Paris, France). 2007;14(3):239–46.
- Ben Salah A, Ben Ismail R, Amri F, Chlif S, Ben Rzig F, Kharrat H, et al. Investigation of the spread of human visceral leishmaniasis in central Tunisia. Transactions of the Royal Society of Tropical Medicine and Hygiene. 2000;94(4):382–6.
- Remadi L, Chargui N, Jimenez M, Molina R, Haouas N, Gonzalez E, et al. Molecular detection and identification of *Leishmania* DNA and blood meal analysis in *Phlebotomus (Larroussius)* species. PLoS Neglected Tropical Diseases. 2020;14(3):e0008077.
